# Evaluating family knowledge about sexual health in patients with severe mental illness: a qualitative study in Iran

**DOI:** 10.1186/s12888-022-03788-4

**Published:** 2022-03-10

**Authors:** Zahra Mirsepassi, Firoozeh Raisi, Zahra Shahvari, Reihaneh FirooziKhojastefar, Seyyed Taha Yahyavi

**Affiliations:** 1 Department of Psychiatry, Roozbeh Hospital, School of Medicine, Tehran University of Medical Sciences, Tehran, Iran; 2 Department of Nursing and Midwifery, Gachsaran Branch, Islamic Azad University, Gachsaran, Iran

**Keywords:** Sexual health, Sex education, Severe mental illness, Family role, And qualitative study

## Abstract

**Introduction:**

Although paying more attention to sex education in patients with severe mental illness is recommended in the literature, the role of families has not been specifically clarified.

**Aim:**

This study aims to explore family knowledge about sexual health in patients with severe mental illness in Iran.

**Methods:**

We conducted a total number of 21 interviews with 4 patients, 5 families, 7 psychiatrists, 1 general practitioner, 2 nurses, and 2 psychologists through purposive sampling. The text was analyzed using conventional qualitative content analysis.

**Results:**

The family knowledge about patients’ sexual health is described in three subcategories: ‘informal sources for knowledge acquisition’, ‘common myths, and ‘inappropriate reaction to the patients’ needs”.

**Conclusions:**

Family sex education should be integrated into a comprehensive rehabilitation program to promote sexual health in patients with severe mental illness. Family members should be aware of the necessity of accurate information about patients’ sexual concerns.

**Supplementary Information:**

The online version contains supplementary material available at 10.1186/s12888-022-03788-4.

## Introduction

The Sexuality Information and Education Council of the United States (SIECUS) believe that sex education is an inalienable right for all human beings [14]. Sex education has sometimes been neglected in patients with severe mental illness (SMI) [4, 28, 30, 33, 34, 40].

According to the literature, paying more attention to sex education in patients with severe mental illness is recommended. Risky behaviours and substance uses are some risk factors that make these patients prone to sexually transmitted diseases (STD) [4, 8, 23, 24, 31, 32, 35, 43]. A recent study in Ethiopia presents some predictors for risky behaviours in these patients including, male gender, illiteracy, bipolarity, history of hospitalization, perceived internal stigma and poor social support [10].

These at-risk patients do not have sufficient knowledge about HIV-related issues [18]. A recent meta-analysis proposed that sexual health need assessment should be integrated into the routine practice [16, 18, 24]. Therefore, health care providers; and nurses should be trained to educate patients and their families [18].

### Family involvement in SMI

Family members are providing informal care to these patients [6, 36]. A study suggests that 50–80% of patients with schizophrenia are in close contact with their relatives in western countries [6]. We should note that there are some differences between some western countries. Northern and southern European countries display this distinction. The family plays a smaller importance in Finland, a northern European country, than it does in Spain, a southern European country [37]. In Iran, as a developing country, most of the patients live with their relatives. Therefore, the role of the family is critical in this context [29]. Another crucial point is that in Iranian culture, sexual concerns appear to be a taboo subject that families are typically hesitant to discuss [1], and this is likely to be exacerbated in families with a patient with serious mental illness.

### Gaps in current family intervention/psychoeducation

The implementation of family involvement in mental health care, particularly for persons with severe mental illness has been emphasized based on convincing reasons. Review of the literature indicates that family interventions may improve social function, self-experienced health and adherence with medication. Moreover, it decreases the frequency of relapse, hospital admissions in patients with severe mental illness [15].

Despite of the fact that many individuals with SMI are intermittently sexually active, and those who are sexually active tend to engage in risky sexual practices, role of the family intervention in this area has not been noted. While, some of these patients have cognitive processing difficulties or are unable to articulate their thoughts, which may interfere with their ability to attend to, understand, or process information in sexual risk reduction interventions [38]. In such a cases of patients with SMI, active role of the family in sex education program cannot be ignored.

### Sexual health needs and risks

Sexuality education and healthcare should be available to everyone. A sexual rights-based approach appears to be especially relevant for potentially vulnerable populations such as those with severe mental illness [21]. Women and young people with mental illnesses have unique demands in terms of sexuality and sexual health education. They could also be more vulnerable towards sexual abuse and exploitation as a result of unequal power balances in relationships and/or early experiences of sexual abuse, increased rates of re-victimization in adulthood [39]. Gordon et al., reported that sexual activity in severe mental illness patients was frequently unplanned, ending in unprotected sex, and that risk perceptions were frequently erroneous, leading in a lack of willingness to reduce sexual risk behaviors [12]. Individuals with severe mental illness are frequently unable to work, results in homelessness and poverty. As a consequence, they cannot afford condoms, lack the privacy to discuss safer sex with partners, and may live in regions where STDs and HIV are prevalent [38]. In general, people with SMIs appear to engage in behaviors that put them at higher risk for HIV and other STDs.

Patients with severe mental illness, as a vulnerable population that engages in high-risk behaviors, should have access to sexual health program education. Family engagement and psychoeducation should include not only general information about the health service, the disease process, relapse symptoms, and medication, but also information regarding the patient’s sexual life. This study aims to explore the knowledge of family members about sexual health in patients with SMI in Iran.

## Material & method

### Design

This qualitative study is part of a research project on designing an educational package about sexual health for patients with SMI.

### Setting

The interviews with the patients or family members were made in a private location in the rehabilitation center of one of the main referral psychiatric hospitals in Tehran, which mostly provide mental health services to consumers with a low socioeconomic status. In addition, the interviews with the health care providers were conducted in their offices or clinic.

### Participants

We used purposive sampling to select the participants. We conducted 21 face-to-face, deep and semi-structured interviews with 4 patients, 5 families, 7 psychiatrists, 1 general practitioner, 2 nurses, and 2 psychologists. The mean age of patients and family members was 33 (17–49) and 50 (29–60) years old, respectively. The mean age of clinicians was 59 (42–70). All of them were Iranian, sexual orientation of all patients were heterosexual, they were in middle social level. Two of patients and 3 family members were diploma, and others had academic education. All of the health care providers were experts in their field. The eligible criteria for patient participants were being over 18 years old and having the diagnosis of SMI in the remission phase. The eligible criteria for family members were being over 18 years old and having a relative with SMI. Patients with the diagnoses of schizophrenia, schizoaffective or bipolar disorder were considered as having a severe mental illness.

Two psychiatrists and a provider with a doctorate in reproductive health care conducted the interviews. The duration of each interview was 60–90 min. An expert psychiatrist in the psychosexual field supervised all the qualitative interviews.

### Procedures

We explained the aim of the research to all of the participants and audio recorded all of the interviews. Nobody refused to participate, and nobody dropped out.

In the interviews, the healthcare provider participants were asked about their general experience regarding sexuality in patients with severe mental illness. Then, the interviewer tried to address experiences that were more related to the study, such as sexual needs in patients with SMI and the role of the family. The patients and family members were asked their opinion about patients’ sexual issues and the necessity to access educational content. Then experiencing any challenges in sexual issues due to the illness, sexual concerns and some more specific domains were addressed (please see the topic guide).

According to the answers, the interviewer used probing and reflective expressions to encourage experiential specificity [25].

The authors suggested a guideline for the interviews. This guideline was used in the first two pilot interviews and corrected according to the team members’ feedback.

### Analysis

After the interview sessions, the collected data were transcribed and analyzed using qualitative conventional content analysis [19]. Coding was used from the first interviews and tested and revised through analysis of succeeding interviews.

Data analysis was inductive. Repeated reading of the extracted codes helped us identify the similarities and differences of textual data, and classify and organize them. Through careful inspection and continuous and advanced data comparison, categories emerged by inductive reasoning [17, 45]. Extracted codes were managed via MAXQDA 10 software.

The researchers were committed to Lincoln and Guba’s four main criteria in a qualitative approach [22].

Two researchers coded the interviews independently. Only one member of the research team did data analysis to enhance interviewer consistency and reliability. The codes were reviewed and discussed in several sessions by all researchers. The researchers tried to obtain an overall sense of the implication of each interview. The results were member checked, and participants confirmed the codes. The results were discussed, and the research team agreed upon all revisions.

The interviews continued until no new information was gained, and then two interviews were done. At this time, the data were considered to be saturated.

### Ethical consideration

This study was performed under the Declaration of Helsinki. Written and oral consent was obtained from all participants, and the Ethics Committee of Tehran University of Medical Sciences approved the study (Ethic’s Code: IR.TUMS.MEDICINE.REC.1395.914).

## Results

Evaluating family members’ knowledge about patients’ sexual health is reported in Table 1.

**Table 1:** Evaluating family members’ knowledge about patients’ sexual health

Category	Subcategory	Codes
Evaluating family members’ knowledge about patients’ sexual health	A. Informal sources for knowledge acquisition	a. Acquaintances b. Family members of other patients c. The media d. Informal social activists’ networks
	B. Common myths	a. The lack of sexual needs in patients b. Feasibility of lifelong abstinence c. Restriction as the only way of sexual health d. Marriage as a treatment for mental illness
	C. Inappropriate reaction to the patient needs	a. Considering the patient as incompetent b. Over-involvement and overprotection c. *Not* considering transient sexual relationships as a risky behavior

We found three subcategories:

a Informal sources for knowledge acquisition

The findings represent the family members’ resources to obtain information about sexual issues in patients with mental illness are usually informal The codes are: ‘acquaintances’, ‘family members of another patient’, ‘the media’, and ‘informal social activists’ networks’.

Some of the statements are:

'*I was talking to other families. There was a mother who was worried about the future of her son. She asked me to introduce someone to take care of her son (she looking for a wife for her son)*' (A 49-year-old sister).

'*We should mention the role of the media and the internet. Sometimes, families get information about sexual issues through searches on websites or virtual networks*' (A 49-year-old clinician).

*'Patients may have virtual friends. Family members should be aware of it. They should help the patients in making decisions in their relationships. Furthermore, patients can search for everything by using the internet. Some of this information is incorrect, and some may be exaggerated. Family members should transfer this information to the clinicians. Clinicians can correct the incorrect beliefs'* (A 55-year-old GP).

b Common myths

This subcategory reflects some common incorrect beliefs of families which are actually exist in general population in our society The codes are:

‘The lack of sexual needs in patients’, ‘feasibility of lifelong abstinence’, ‘restriction the only way of sexual health’, and ‘marriage as a treatment for mental illness’.

Some of the statements are:

‘*The attitude of parents has a major role. They may ignore the patients’ sexual nee*d’ (A 42-year-old psychiatrist). Moreover, a father told although my son is a patient but he is really pious so could control his sexual needs (A 51-year-old father).

‘*Sometimes family members believe that marriage is a treatment for mental illness*’ (A 45-year-old nurse). One of the participants told us, marriage could improve her son’s disorder, especially with satisfying his sexual needs. (A 49-year-old sister).

*‘Some families believe that the only solution for maintaining sexual health in their relatives is to restrict them,* (A 65-year-old psychiatrist). Additionally, a mother told us that she knows her daughter has sexual needs but she must learn control herself completely (A 50-year-old mother). Moreover, some families are worried about giving sex education to the patients. For example, a mother told us that her daughter knows nothing about sexual issues and talking with her causes sexual concern (A 50-year-old mother).

iii Inappropriate reaction to the patients’ needs

This subcategory suggests that some family members may present some inappropriate reactions when faced with a patient’s sexual issues. The codes are:

Considering the patient as incompetent’, and ‘need to over-involvement and overprotection’. ‘Not considering transient sexual relationship as a risky behavior’.

Following, one of the clinician’s statements is presented:

‘*In the remission phase, patients can manage their sexual needs. Patients need to be equipped with appropriate skills. Skills training should be provided in a persistent manner*’ although, most of the families think that the patients are completely incompetent to manage their sexual behaviors (A 55-year-old GP).

'*In the remission phase, we should provide services such as booster educational sessions and address sexual issues*' (A 65-year-old psychologist).

'*Family members are worried about their loved one. So, they may become more controlling or overprotective. This approach may build a high emotional expression environment*' (A 37-year-old patient).

*'My Mother had an inappropriate reaction when I mentioned my sexual problems, she overprotects me and it is not acceptable*' (A 37-year-old patient).

*Also, 'Some family members believe that having transient sexual relationship especially in male patients could be considered as a solution for patients' sexual needs. They do not consider it as a risky behavior'* (A 55-year-old GP).

Finally, sometimes patients want to talk with one of the family members about their sexual concerns so the families should be educated in this regard. For example, they might become frustrated because of sexual dysfunction and they need to be assured that they can share their emotions with family members’ (A 65-year-old psychologist).

## Discussions

In this qualitative study that aimed to explain the knowledge of SMI’s family about sexual health, three main categories were emerged. “Selecting an informal source for knowledge acquisition” explain that SMI’s family does not refer to reliable scientific sources for obtaining sexual information or these sources are not readily available to them. “Believing in common myths” explains that there are common misconceptions in SMI’s families, such as they do not have sexual needs, they should abstain completely over the life, marriage is a treatment for psychiatric illness or transient sexual relation is not risky. “Inappropriate reaction to the patients’ needs” explains that when patients’ families encounter the patient’s sexual issues, they react emotionally and irrationally, such as blaming them or trying to over-care or control them.

Although most studies have emphasized the importance of sex education in SMI, the role of families should be clarified in more details. This study has conducted in Iran, a developing country which is located in Middle East with a collectivistic and Islamic culture, and relatively poor social welfare and weak systematic social support. Therefore, it is obvious that in this society how prominent the role of SMI’s families could be in managing the patients. Family members usually experience a high amount of burden [9] and stigma according to caring for the patients [2, 44].

Families may feel ashamed to talk about patients’ sexual issues with clinicians. On the other hand, clinicians also may feel uncomfortable and unequipped in sexual history taking. These feelings in families and clinicians may lead to ignore the sexual issues in patients [5, 30]. Therefore, education about clinicians and SMIs’ families role in improving patient’s sexual health is necessary (Eddie [26]; Edward [27, 28, 30]).

In societies that families are mostly accountable for SMIs’ care, the impact of their attitudes and actions on patients’ sexual health is very high [29, 37]. In one hand, there is no systematic sexual health education even for general population in most Islamic country including Iran, and on the other hand general knowledge of SMIs’ family for taking care of patients and themselves is negligible in middle east [2]. This fact is in line with our findings that SMIs’ family get necessary information from unreliable and informal sources. Therefore, a two way movement should take place to change the condition. Up down attitude about the necessity of sexual health education should be upgraded and bottom up request for such education should be stimulated. This bidirectional movement is highly important for SMIs’ families [2, 41]. In addition of receiving information and advice from health care providers, SMIs’ families need more support from other resources to reduce the burden [42].

Therefore, family education is recommended to improve sexual health outcomes in patients with SMI [13]. Clinicians should correct common myths in family education sessions. Although SMI patients need to be supported, family members should be aware of the possibility of lapse in over-controlling and negative consequence of high emotional expression [3]. In the family members view, the patient may consider incompetent. This attitude may create overprotection and create a negative emotional atmosphere [3]. As a result, these patients may experience stigma and discrimination [11]. Family members are in close contact with the patients. They should accept the mental illness in their loved ones, but being a psychiatric patient is not synonymous with incompetence. These patients are interested to learn about sexual health [20]. Although they may lose some of their skills and capabilities, they should be educated about sexual issues [7] (Edward [27]). They can manage sexual issues in remission periods.

Family members should be aware of the risk of STDs, AIDS, and unwanted pregnancies. Although some patients should be supervised by families, it is necessary to be aware of the negative consequence of high emotional expression. Authors suggest that a good relationship between the patient and families and a sense of trust can reduce family concerns. An atmosphere in which the patient feels secure and comfortable to talk about sexual concerns; and find his/her family member, a good listener.

### Implications in the practice

Mental health care providers, including nurses, General Practitioners, clinical psychologists and psychiatrists should be trained to assess the sexual health needs of these patients alongside asking family members about their concerns. Family education about patients’ sexuality, should be integrated into a comprehensive rehabilitation program.

The authors suggest that according to cultural issues in, family engagement is helpful to assess patients’ sexual health. The results highlighted the importance of sex education for families and the necessary topics. The authors recommend planning for adding a sex education component to the whole package of family psycho-education for SMI patients. Therefore, family education is recommended to improve sexual health outcomes in patients with SMI [13]. Clinicians should correct common myths and misbeliefs in family education sessions.

Family members should be aware of the risk of STDs, AIDS, and unwanted pregnancies. Although some patients should be supervised by families, it is necessary to be aware of the negative consequence of high emotional expression.

Authors suggest that a good relationship between the patient and families and a sense of trust can reduce family concerns. An atmosphere in which the patient feels secure and comfortable to talk about sexual concerns; and find his/her family member, a good listener.

### Limitations

The patients and family members were selected from the clients of Roozbeh Day Centre using purposive sampling. Roozbeh Hospital is a tertiary center located in Tehran, the capital city of Iran, and the patients and families referred to this center might not representative of all peers. Although we emphasized confidentiality before starting the interviews, it is probable that some participants have not feel comfortable disclosing their experiences according to the sensitivity of the topic.

### Recommendation for future research

Evaluation of the effect of family sexual health education in reducing family burden, promoting communication with the patients and reducing sexual risk behaviors is recommended for future research.

## Conclusions

Clinicians should be aware of the sexual needs of these patients. They should ask about sexual health concerns from the patients and their families. They should correct the incorrect beliefs of family members, and recommend accessible resources to learn about the sexual issues in these patients.

## Supplementary Information

**Additional file 1.**

## Data Availability

The datasets used and/or analyzed during the current study are not publicly available due to the confidentiality of the patients and their interviews but are available from the corresponding author on request.
