# Supplementary material for: Evaluating family knowledge about sexual health in patients with severe mental illness: a qualitative study in Iran
Source: BMC Psychiatry. 2022 Mar 10;22:174. doi: 10.1186/s12888-022-03788-4 (PMC8908587; doi:10.1186/s12888-022-03788-4)
Supplement: Supplementary file 1 — Additional file 1. [file 12888_2022_3788_MOESM1_ESM.docx]

**Topic guide for the interviews with experts**

- What do you think about sexual health in patients with severe mental illness? Why?
- What do you think about the role of families in sexual health in patients with severe mental illness? Why?
- In your opinion, is it necessary to design educational content for sexual health for their families? Why?
- What is your recommendation for the educational content? Why?
- The second part of the interviews focus on the educational topics:
- Is it necessary for the families to be aware of sexual needs in patients with severe mental illness? Why?
- Is it necessary for the families to be aware of different contraceptive methods? Why?
- Is it necessary for the families to be aware of medications side effects? Why? What about sexual side effects? Why?
- Is it necessary for patients and families to be aware of the risk of STDs? Why?
- Is it necessary for patients and families to be aware of the role of relational problems on sexual health? Why?

**Topic guide for the interviews with patients**

- What do you think about your sexual issues?
- Do you feel any necessity to access to educational content about sexual issues?
- Do you experience any challenges in sexual issues due to the illness?
- Do you experience any sexual side effects due to medications?
- Is there anybody to talk to when you have some concerns about sexual issues? What are the characteristics?
- The second part of the interviews focus on the educational topics:
- Do you have any concerns about the possibility of pregnancy in a sexual relationship?
- What do you know about contraceptive methods? How do you obtain this information?
- What do you know about medications sexual side effects? How do you obtain this information?
- What do you know about STDs? How do you obtain this information?
- Do you feel comfortable speaking to clinicians about sexual issues? Do you have any experience in avoiding talking or neglecting these concerns? What are the reasons?

**Topic guide for the interviews with family members**

- What do you think about sexual issues in patients with severe mental illness?
- Do you feel any necessity to access educational content about sexual issues in patients with severe mental illness?
- What is your recommendation for the educational content? Why?
- Do you experience any challenges in the patient`s sexual issues due to the illness?
- Do you experience any challenges about sexual side effects due to medication in the patient?
- Does the patient talk to you about sexual concerns? Why?
- The second part of the interviews focus on the educational topics:
- In your opinion, do these patients experience sexual needs? Why?
- Do you have any concerns about the possibility of pregnancy in your loved one?
- What do you know about contraceptive methods? How do you obtain this information?
- What do you know about medications sexual side effects? How do you receive this information?
- What do you know about STDs? How do you receive
- this information?
- Do you feel comfortable speaking to your loved one or clinicians about sexual issues? Do you have any experience in avoiding these concerns? Why?
